# Pre-Operative Mechanical Bowel Preparation Does Not Affect the Impact of Anastomosis Leakage in Left-Side Colorectal Surgery—A Single Center Observational Study

**DOI:** 10.3390/life14091092

**Published:** 2024-08-30

**Authors:** Ludovít Danihel, Marian Cerny, Ivor Dropco, Petra Zrnikova, Milan Schnorrer, Marek Smolar, Miloslav Misanik, Stefan Durdik

**Affiliations:** 1 3rd Surgical Clinic, Faculty of Medicine, Comenius University in Bratislava, 814 99 Bratislava, Slovakia; schnorrermi@yahoo.com; 2 Surgical Department, Bory Penta Hospitals, 841 03 Bratislava, Slovakia; 3 Klinik für Allgemein-, Viszeral-, Thorax-, Adipositas-, Gefäß-und Kinderchirurgie, 94032 Passau, Germany; doktor.cerny@gmail.com; 4 Klinik und Poliklinik für Chirurgie, Universitätsklinikum Regensburg, 93053 Regensburg, Germany; ivordropco@web.de; 5 Medicalen, 036 01 Martin, Slovakia; petrazrnikova@medicalen.sk; 6 Clinic of General, Visceral and Transplant Surgery, Jessenius Faculty of Medicine, Martin, Comenius University in Bratislava, 813 72 Bratislava, Slovakia; smoly2002@hotmail.com (M.S.); misanik3@uniba.sk (M.M.); 7 Department of Surgical Oncology, Faculty of Medicine, Comenius University in Bratislava, 813 72 Bratislava, Slovakia; stefan.durdik@ousa.sk

**Keywords:** mechanical bowel preparation, colorectal surgery, anastomosis leakage, antibiotics, safety, surgical site infection

## Abstract

Despite rapid advances in colorectal surgery, morbidity and mortality rates in elective gastrointestinal surgery play a significant role. For decades, there have been tempestuous discussions on preventative measures to minimize the risk of anastomotic dehiscence. When mechanical bowel preparation before an elective procedure, one of the key hypotheses, was introduced into practice, it was assumed that it would decrease the number of infectious complications and anastomotic dehiscence. The advancements in antibiotic treatment supported the concomitant administration of oral antibiotics and mechanical bowel preparation. In the prospective study conducted at our clinic, we performed left-side colorectal procedures without prior mechanical preparation. All patients enrolled in the study underwent the surgery and were observed in the 3rd Surgical Clinic, Faculty of Medicine, Comenius University in Bratislava, Slovakia, from January 2019 to January 2020. As a control group, we used a similar group of patients with MBP. Our observed group included 87 patients with tumors in the left part of their large intestine (lineal flexure, descendent colon, sigmoid colon, and rectum). Dixon laparoscopic resection was performed in 26 patients. Sigmoid laparoscopic resection was performed in 27 patients. In 12 patients, the procedure was started laparoscopically but had to be converted due to adverse anatomical conditions. The conservative approaches mostly included Dixon resections (19 patients), sigmoid colon resections (5 patients), left-side hemicolectomies (6 patients), and Miles’ tumor resections, with rectal amputation (4 patients). Our study highlighted the fact that MBP does not have an unequivocal benefit for patients with colorectal infection, which has an impact on the development of anastomotic dehiscence.

## 1. Introduction

Infectious complications in colorectal surgery are frequent problems, despite the attempt to reduce surgical site infection—SSI (5.4–22.4%)—and anastomotic dehiscence, the incidence rate of which remains at 2–10%. The risk of anastomotic dehiscence increases in patients with low rectal resections or complex inflammatory diseases [1,2]. Initial doubts about the need for mechanical bowel preparation were identified when improved well-being was observed in patients who underwent urgent gastrointestinal surgical procedures. Several studies have presented similar outcomes of surgical procedures, including complications, regardless of the form of pre-operative preparation [3]. Apart from that, in pre-operative preparation, there are no standardized procedures, so each clinic follows its own routine practices. Despite a high number of studies with ambiguous results of preferring MBP available since 1990 [4,5], when MBP was introduced into practice, the key hypothesis assumed that intraluminal content, feces, would be reduced, and thus minimize the risk of anastomotic dehiscence and incidence of infection (SSI). Secondary benefits included the possibility of performing peri-operative tumor palpations and peri-operative colonoscopies. As opinions on MBP have been changing over time, regimens and agents used for bowel preparation have been developed as well. Dietary restrictions, even fasting, and colon lavage were the original framework of pre-operative preparation of the large intestine. However, the discomfort of the patient related to clysters and laxatives and the threat of non-adequate low-calorie intake prior to exacting surgical procedures, as well as several days of pre-operative hospitalization, during which basic food is served, have been identified as unimportant and expensive. Then, an orthograde colon lavage with an intake of a large volume of saline was used [6].

Mannitol (a type of sugar alcohol used as a sweetener) compared to saline has been found to be a great lavage agent with minimal side effects on the human body. A case report and the fear of explosion resulting from the use of electrocautery during the surgery prevented global acceptance of this agent for the pre-operative preparation of the large intestine [7].

Polyethylene glycol (PEG) lavage solution was introduced for the first time in 1980 [8]. Several studies have confirmed its safety, efficacy, and tolerability compared to conventional regimes of bowel preparation [9]. When a PEG regime was used, changes in the mucosa in the intestinal wall were observed, in particular, the loss of surface mucus and epithelial cells as well as inflammatory changes [10].

Despite a large number of scientific research studies on MBP in colorectal surgery, there is a question of whether MPB is necessary at all. Initial doubts about the necessity of performing MBP were published in a study by Hughes in 1972 [11]. The counter-theory claims that the routinely performed mechanical bowel preparation is no longer recommended in the era of antibiotic therapy because there is a risk of developing electrolyte disbalance such as hypokalemia and hypocalcemia, especially in older adults. At the same time, the dilution of feces is believed to increase the probability of leakage and contamination by feces [12]. Also, the ERAS protocol questions its importance [13]. The National Institute of Health and Clinical Excellence (NICE) in the United Kingdom does not recommend performing MBP anymore, in order to decrease SSIs and the risk of anastomotic dehiscence [14]. Various meta-analyses have not shown any significant benefit of MBP for patients with an elective colorectal procedure when compared to patients who did not undergo it [15,16]. Nowadays, intravenous antibiotic prophylaxis at the beginning of anesthesia administration is considered the standard procedure in elective colorectal surgery [17]. Opinions on this topic vary widely in clinical practice, not only between health professionals in different countries and towns but also between those working at the same clinic, which indicates that the approach to this procedure still remains ununified. 

Our study analyzes the incidence of anastomotic dehiscence and early post-operative complications in patients after an elective left-side surgical procedure without mechanical bowel preparation.

## 2. Methods

All patients enrolled in the study underwent the surgery and were observed in the 3rd Surgical Clinic, Faculty of Medicine, Comenius University in Bratislava, Slovakia, and Merciful Brothers University Hospital in Bratislava from January 2019 to January 2020. They were provided detailed information about the type of study and gave their consent to take part in it. 

Our observed group included 87 patients with tumors in the left part of their large bowel (lienal flexure, descendent colon, sigmoid colon, and rectum). The pre-operative bowel preparation we prefer starts at home. It includes a low-residue home-made diet, which the patient eats for 5 days before the scheduled hospital admission for the procedure is performed (Table 1). The composition of allowed foods and the way of their preparation is described in Table 1. After hospital admission, one day before the procedure, the patient ingests liquids only. In the evening before the day of the procedure, a “large” clisma (1000 mL hot water + 150 mL trisodium phosphate) was administered. In the morning before the procedure, a “smaller” clisma (50 mL hot water + 100 mL trisodium phosphate) was administered.

Shortly before the surgical procedure, all patients were administered i.v. prophylactic antibiotics (Vulmizolin 2 g + Metronidazol 500 mg), currently approved by the antibiotic committee in our hospital. On the day of the surgery, 2 h before the procedure, the patients were advised to drink 200 mL of sweet tea or fruit juice without flesh.

An important part of our pre-operative preparation approach is also a correct triage of the patients by their malnutrition risk (Table 2). Pre-operative administration of a nutridrink is not necessary for low-risk patients. In addition to the recommended diet, patients with moderate and high risks are administered 1–2 nutridrinks a day. All patients in both groups had a low malnutritional risk; therefore, consumption of nutridrinks prior to the surgical procedure was not necessary.

In the group of our interest, we observed the type of surgical procedure, the type of anastomosis performed (hand-sewn anastomosis or stapled anastomosis), the degree of conversion from laparoscopy to laparotomy, and morbidity and mortality rates. We evaluated early post-operative complications by their type (wound complications, anastomotic leakage, and need for re-surgery). To obtain a control group, in order to evaluate our results objectively, we compared our group of patients with a group of patients undergoing mechanical bowel preparation, with comparable peri- and post-operative risks as well as similar comorbidities.

## 3. Results

In total, 87 patients, 54 men and 33 women, were enrolled in our study with an average age of 64.02 years (SD—11.31).

The inclusion criteria included verified cancer of the left colon, without previous oncological treatment, and without signs of ileus.

We excluded patients with insufficient compliance from the work. Dixon laparoscopic resection was performed in 26 patients. Sigmoid laparoscopic resection was performed in 27 patients. In 12 patients, the procedure was started laparoscopically but had to be converted due to adverse anatomical conditions. The conversion rate was 18.46%. The conservative approaches mostly included Dixon resections (19 patients), sigmoid colon resections (5 patients), left-side hemicolectomies (6 patients), and Miles´ tumor resections with rectal amputation (4 patients). In 75 cases (86.2%), the anastomosis was performed by a circular stapler. In the rest of the cases (9.19%), the anastomosis was performed by hand-sewing.

Five patients from the other observed group experienced anastomotic leakage confirmed by a CT scan. One patient needed re-surgery. In four cases, a post-operative increase in sanguinolent waste to drain occurred. Conservative therapy was successful in all of these cases (Table 3). Post-operative complication rates of the patients were analyzed according to the Clavien–Dindo complication grade, and when all groups were evaluated, major complications (3b and above) were seen in three patients.

Wound complications in the form of surgical wound seroma occurred in two patients (2.3%). Their treatment included drainage and re-dressing (Table 4).

Our control group consisted of 98 patients, 63 men and 35 women, with an average age of 65.12 years (SD—11.71). Laparoscopic resection was performed in 73 patients and conservative therapy in 22 patients. Conversion from laparoscopy to laparotomy was needed for four patients.

All operations were performed by experienced colorectal surgeons (minimum colorectal resections in a year—30).

We did not find a significant difference between the two groups, which supports the hypothesis that MBP is not decisive in relation to the occurrence of anastomotic leakage in left-sided colorectal procedures.

## 4. Discussion

Pre-operative mechanical bowel preparation was introduced to visceral surgery more than 120 years ago. The primary reason for this was a high rate of infectious complications during elective colorectal surgery. It was even believed that besides the surgeon´s experience, the outcomes of the surgical procedure are influenced by the degree of bowel clearance. Since then, mechanical bowel preparation methods have found their place in a wide range of procedures. The discovery of antibiotics and their combination with MBP has decreased the number of peri-operative infections.

In the 1970s in the 20th century, MBP became a commonly used and accepted technique by surgeons. During this period, a wide range of various methods had been developed, ranging from dietary restrictions to lavages with large volumes of saline solution administered by a nasogastric tube [18,19].

Polyethylene glycol solutions used up to date were introduced shortly after this period as a better option to former regimes. Benefits included better tolerability by the patient and minimal systemic absorption with a decreased disruption of the inner environment of the human body and electrolytes, and their use was less time-consuming. In 1972, Hughes was the first to question the role of MBP. He claimed that the threat of sepsis and complications related to anastomoses is not higher in the unprepared bowel, and thus bowel preparation is not important at all [11]. In 1987, Irving and Scrimgeour confirmed this evidence in their publication consisting of a series of case reports of patients without bowel preparation and without anastomotic complications [20]. Evidence for this theory was observed mainly in traumatological patients with a low percentage of post-operative infections after urgent intestinal procedures without prior bowel preparation, which also resulted in the re-evaluation of MBP [21,22].

Thanks to advances in surgical techniques, instruments, and post-operative care, the well-being of patients who underwent the urgent procedure has improved. This raised the question of whether MBP is necessary in the elective procedure at all. The negative impact of MBP on the anastomotic dehiscence rate and the insufficient effectivity of mechanical preparation and its application have decreased its use in clinical practice [23]. The results of randomized trials and meta-analyses conducted in recent years helped us to understand that mechanical bowel preparation does not have any benefit for post-operative outcomes [24,25].

The pre-operative and peri-operative administration of oral and/or venous antibiotics prior to MBP has attained a more significant role. The number of randomized multicentric studies evaluating various types of preparation and their combination has increased. Currently, it is known that the incidence rate of SSI in elective colorectal surgery is approximately 11.4% (5–22%). Based on the doubts about the role of MBP, the first randomized clinical trials comparing the procedures with and without MBP were conducted and published in Latin America and Europe in the 1990s [5,26,27], which were followed by other studies. The results were limited by the variability of the methods and the inclusive criteria applied. The most well-known one was a critical analysis of the key question comparing the procedures with and without MBP in a Cochrane Library Systemic Database review published in 2003, updated in 2005 and 2011 [25]. Surprisingly, statistical analysis revealed more insufficient anastomoses (AL) in the MBP group (6.2%) compared to the group without MBP (3.2%; *p* = 0.003). The authors concluded that there is no clear evidence of MBP being related to the decreased occurrence of AL after elective intestinal resections.

However, a system meta-analysis conducted in 2022 [28] and a publication by Toh et al. [29] pointed out that the administration of oral antibiotics was associated with a non-significant decrease in anastomotic dehiscence (AL). A summary of all the results of the randomized clinical trials revealed that the combination of oral and venous antibiotics administered to patients undergoing an elective colorectal surgical procedure decreased the incidence rate of SSI. The most recent meta-analysis conducted by Woodfield et al. from New Zealand was published in *JAMA Surgery* in 2022 [28] (Table 5). They summarized data from all randomized clinical trials (RCTs) conducted before 2021 available in Medline, Embase, Cochrane, and Scopus databases, which aimed to match various strategies of bowel preparation with post-operative outcomes. 

Primary results were focused on the incidence rates of SSI and AL. Secondary results included other infections, mortality rate, ileus, and adverse effects of the preparation. In total, 8377 patients from 35 RCTs were identified. The combination of methods is shown in Table 5.

Important contributions were brought up by the MOBILE trial, which compared a group with MBP and antibiotic prophylaxis with a group without mechanical bowel preparation. Especially, the results of left-side procedures are important for the description of MBP and future trends. According to this trial, SSI in left-side procedures reached 6% in patients with MBP and 10% in patients without it (OR = 0.57, 95% CI = 0.18–1.82; *p* = 0.338) [30].

The most important factor in the morbidity and mortality rate in patients undergoing a colorectal procedure is the incidence of anastomotic dehiscence. Several guidelines present a dehiscence of up to 8% as acceptable. Various trials and meta-analyses confirmed that MBP does not have a significant benefit on any of the defined primary goals [31,32,33]. These works are long-term trials published over the last 10 years, thus supporting the evidence that they are not tendentious works, but research based on real data [34,35] (Table 6 and Table 7).

It is important to consider the role of the microbiome, which has not been sufficiently described so far. It is known that the microbiome is significantly influenced by MBP and oral antibiotic prophylaxis, which results in post-operative complications. Alverdy et al. supported this theory by demonstrating the disruption of the fine balance between pathogen proliferation and the natural suppression of normal microflora rearrangement [48]. 

The microbiome has a very wide range of health benefits for the host [49], and disrupted intestinal ecology influences both the efficacy and toxicity of adjuvant chemotherapy [50]. The importance of the microbiome therefore varies, and it is unclear what harm may result from attempting to eradicate the entire microbiome of the large intestine. The diversity of intestinal microflora is considered a key component of health, and therefore, the relevance of eradicating the entire flora to benefit surgical outcomes is questionable.

To date, no international or national surgical association has approved in their guidelines a standard scheme of pre-operative preparation of the large intestine prior to the elective colorectal surgery, nor have recommendations been made to abandon mechanical bowel clearance alone. Similarly, Canadian and Australian guidelines do not consider it necessary [51] (Table 8).

The only clearly defined recommendation is part of the ERAS (enhanced recovery after surgery) concept, which claims that mechanical bowel preparation alone has no clinical benefits, may cause dehydration and discomfort, and should not be routinely performed in colorectal surgical procedures, but may be used in rectal surgical procedures.

Our study supported the evidence from these international studies. We believe that MBP in elective colorectal surgery is more than just a questionable approach and should not be performed on a regular basis because only some patients can benefit from it. Of course, in situations when a NOTES operation or intracorporal anastomosis is made, MBP is profitable for the patient because the contamination of the intraperitoneal space is minimized due to this approach. Still, the new trends do not recommend a routine MBP. The discussion is still not over, and it will probably take years to obtain a high recommendation from the guidelines. 

Surgical site infections are an Achilles’ heel condition after colorectal surgery. Within the framework of the ERAS protocols, mechanical and oral antibiotic bowel preparations have been abandoned for decades. However, the rate of anastomotic leakage, one of the most feared complications after colorectal surgery, has not changed. Contrary to dogma and popular belief, data from patients who did not undergo mechanical bowel preparation were analyzed and discussed with the current literature in this study. Surgical site infections, post-operative mortality, intra-abdominal collection rates, and anastomotic leakage were similar [52].

Currently, optimized peri-surgical management should be mandatory in elective surgical procedures because it improves the post-operative recovery of a patient and decreases the morbidity rate and infectious complications [53].

## 5. Conclusions

The approach to MBP in elective colorectal surgery remains a widely discussed topic. Despite the wide range of specialized studies conducted, bowel preparation using MBP is still unclear. Although there was initial enthusiasm for MBP, gradually its role began to be questioned. Recent studies have strongly supported the evidence that not all patients can benefit from MBP. On the contrary, they confirmed that MBP can result in an increased number of complications. The data from various studies have shown that MBP does not play a role in the risk of developing anastomotic dehiscence. However, the future of this hypothesis depends on professional associations.

## Figures and Tables

**Table 1:** A summary of the low-residue diet.

Type of foods	Lean meat (veal, beef, pork, chicken), fish filet Soups, butter, oil, milk, dairy products Mashed potatoes, thick sauces Rise, soft-boiled egg Fine biscuits, crispbread, stale bakery products, fruit compotes Early harvest vegetables (carrots, cauliflower, kohlrabi, celery, parsley, spinach) All beverages
Way of preparation	All kinds of meat should be ground and liquidized immediately once they are cooked. The mashed potatoes and thick sauces should be liquidized. Vegetables should be soft-cooked.

**Table 2:** The stages of malnutrition risk in patients before a colorectal surgical procedure.

Low risk	Loss of weight >5% in last 2 months or oral intake <50–75% compared to the previous period
Moderate risk	Loss of weight >5% in last 2 months or BMI 18.5–20.5 as well as worsening of patient´s overall condition and oral intake <25–50% compared to the previous period
High risk	Loss of weight >5% in last 2 months (approx. 15% in 3 months), BMI < 18.5 as well as worsening of patient´s overall condition and oral intake <0–25% compared to the previous period

**Table 3:** A summary of the left-side procedures without and with MBP.

Surgical Procedure	(*n* = 87)	(*n* = 98)
*Conservative surgery*	30 (34.48%)	22 (22.45%)
*Laparoscopic surgery*	53 (60.92%)	73 (74.49%)
*Conversion from laparoscopy*	12 (22.64%)	4 (5.48%)
*Other*	4 (4.6%)	3 (3.06%)
*Hand-sewn anastomosis*	8 (9.19%)	10 (10.2%)
*Stapled anastomosis*	75 (86.21%)	85 (86.74%)

**Table 4:** A summary of complications in patients with a left-side procedure without and with MBP.

Complications	(*n* = 87)	(*n* = 98)
*Wound complications*	2 (2.30%)	1 (1.02%)
*-seroma*	2 (2.30%)	0
*-SSI*	0	1 (1.02%)
*Bleeding*	4 (4.60%)	0
*Anastomotic leakage*	5 (5.74%)	1 (1.02%)
*Colon*	2	0
*Rectum*	3	1
*Ileus condition*	0	0
*Intra-abdominal infection*	0	0
*Total rate of conversions*	11 (12.64%)	1 (1.02%)
*Re-surgery*	1 (1.15%)	1 (1.02%)

**Table 5:** The combinations of methods of bowel preparation published by Woodfield et al. in *JAMA Surgery* [28].

	OR (95% CI ^a^)	
Treatment	IV + OA	MBP + IV + OA
OA alone	0.15 (0.06–0.33) ^b^	0.19(0.08–0.43) ^b^
MBP + OA	0.10 (0.04–0.25) ^b^	0.14 (0.07–0.31) ^b^
MBP + IVB + OA	0.18 (0.08–0.41) ^b^	0.25 (0.12–0.51) ^b^
MBP + IV	0.22 (0.12–0.40) ^b^	0.31 (0.20–0.48) ^b^
IV alone	0.27 (0.15–0.50) ^b^	0.38 (0.24–0.62) ^b^
IV + E	0.26 (0.11–0.63) ^c^	0.37 (0.17–0.81) ^d^
MBP + IV + OA	0.71 (0.41–1.21)	NA
IV + OA ± E	NA	1.41 (0.83–2.42)

Abbreviations: E—enema, IV—intravenous antibiotics, IVB—inadequate IV antibiotics, MBP—mechanical bowel preparation, OAs—oral antibiotics. a—an OR less than 1 means that surgical SSI is less likely after the treatment in the column compared with the treatment in the corresponding row. For example, an OR of 0.5 means that the occurrence of an SSI is half as likely for the treatment in the column than for the treatment in the corresponding row. b—*p* < 0.001. c—*p* < 0.01. d—*p* = 0.01.

**Table 6:** A summary of the trials analyzing patients with and without MBP [34].

First Author	Study Design	Year	No. of Patients	Population	MBP	Non-MBP	*p*-Value	MBP	Non-MBP	*p*-Value
Zmora [36]	Randomized prospective trial	2003	380	Elective colon and rectal surgery with primary anastomosis	12/187 (6.4)	11/193 (5.7)	NS	7/187 (3.7)	4/193 (2.1)	NS
Bucher [37]	Randomized clinical trial	2005	153	Elective left-sided colorectal surgery with primary anastomosis	10/78 (12.8)	3/75 (4.0)	0.070	5/78 (6.4)	1/75 (1.3)	0.210
Santos [5]	Prospective randomized trial	1994	149	Elective colorectal surgery	17/72 (23.6)	9/77 (11.7)	<0.050	7/72 (9.7)	4/77 (5.2)	0.520
Jung [31]	Multicenter randomized clinical trial	2007	1343	Elective open colon surgery	54/686 (7.9)	42/657 (6.8)	NS	13/686 (2.3)	17/657 (2.6)	NS
Fa-SI-Oen [38]	Multicenter randomized clinical trial	2005	250	Elective colon surgery	9/125 (7.2)	7/125 (5.6)	0.610	7/125 (5.6)	6/125 (4.8)	0.780
Pineda [39]	Meta-analysis and review	2008	4601 (13 trials)	Elective colorectal surgical resection	227/2304 (9.9)	201/2297 (8.8)	0.155	97/2304 (4.2)	81/2297 (3.5)	0.206
Slim [40]	Meta-analysis and review	2004	1454 (7 trials)	Elective colorectal surgery	53/720 (7.4)	42/734 (5.7)	0.175	39/701 (5.6)	23/708 (3.2)	0.032
Guenaga [25]	Meta-analysis and review	2011	5805 (18 trials)	Elective colorectal surgery	223/2305 (9.7)	196/2290 (8.6)	NS	101/2275 (4.4)	103/2258 (4.5)	NS

**Table 7:** A summary of the trials analyzing patients with and without MBP [35].

Study	# of Patients	MBP Agent	Anastomotic Leaks with MBP (%)	Anastomotic Leaks without MBP (%)	*p* Value	Wound Infections with MBP (%)	Wound Infections without MBP (%)	*p* Value
Brownson et al., 1992 [26]	179	PEG	11.9	1.5	0.03	5.8	7.5	0.77
Santos et al., 1994 [5]	149	Mineral oil, agar, and phenol-phthalein; mannitol	10.4	5.3	0.34	23.6	11.7	0.08
Burke et al., 1994 [27]	169	Sodium picosulfate	3.7	4.6	1	4.9	3.4	0.71
Fillman et al., 1995 [41]	60	Mannitol	8.7	4.3	1	3.3	6.7	1
Tabusso et al., 2002 [42]	47	Mannitol or PEG	20.8	0	0.04	8.3	0	0.49
Miettinen et al., 2003 [43]	267	PEG	3.8	2.5	0.72	3.6	2.3	0.72
Bucher et al., 2006 [37]	153	PEG	6.4	1.3	0.21	12.8	4	0.07
Ram et al., 2005 [44]	329	NaP	0.6	1.3	1	9.8	6.1	0.22
Fa-Si-Oen et al., 2005 [38]	250	PEG	5.6	4.8	0.78	7.2	5.6	0.79
Zmora et al., 2006 [45]	249	PEG	4.2	2.3	0.48	6.7	10.1	0.36
Pena-Soria et al., 2007 [46]	97	PEG	8.3	4.1	0.05	12.5	12.2	1
Jung et al., 2007 [31]	1343	PEG NaP Enema	1.9	2.6	0.46	7.9	6.4	0.34
Contant et al., 2007 [47]	1354	PEG + bisacodyl or NaP	4.8	5.4	0.69	13.4	14.0	0.75

**Table 8:** Current recommendations for the use of MBP and OA in elective colorectal surgery.

Guideline	Year of Publication	Mechanical Bowel Preparation (MBP)	Oral Antibiotic Prophylaxis (OAP)	Perioperative Intravenous (IV) Antibiotics
World Health Organisation (WHO)	2018	(x)	(x)	(x)
National Institute of Clinical Excellence (NICE)	2019			(x)
American Society of Colon and Rectal Surgeons (ASCRS)	2019	(x)	(x)	(x)
Association of Coloproctology of Great Britain & Ireland (ACPGBI)	2017			(x)
Australian Cancer Council	2018			(x)
Japan Society for Surgical Infection	2021	(x)	(x)	(x)

(x) indicates that there were recommendations made by individual associations.

## Data Availability

The original contributions presented in the study are included in the article, further inquiries can be directed to the corresponding author.
